# Exploring positive welfare measures: preliminary findings from a prototype protocol in loose housing dairy cattle farms

**DOI:** 10.3389/fvets.2024.1368363

**Published:** 2024-06-27

**Authors:** Silvana Mattiello, Stefania Celozzi, Federica Manila Soli, Monica Battini

**Affiliations:** Department of Agricultural and Environmental Sciences – Production, Landscape, Agroenergy, University of Milan, Milan, Italy

**Keywords:** dairy cows, positive affects, animal emotions, behavior, animal welfare, housing, feeding, welfare protocol

## Abstract

**Introduction:**

Following the increasing interest about the development of indicators of positive welfare and affective state in farm animals, the aim of this research is to present some preliminary results on the application of a prototype protocol based exclusively on positive welfare measures and to suggest potential benefits that can promote positive welfare.

**Methods:**

The protocol was applied in 20 loose housing dairy cattle farms (6 on deep litter with straw, 14 in cubicles) and included only indicators of positive welfare and emotional states: feeding and resting synchronization, rumination during resting, comfortable lying postures, no visible eye white, relaxed ear postures, percentage of cow contacts with humans in the Avoidance Distance test. Potential benefits in terms of housing, feeding and management were then related to these variables (Mann-Whitney U test). Qualitative Behavior Assessment (QBA) was also carried out and analyzed by Principal Component Analysis to explore the effect of factors that were not evenly distributed in our sample (number of feed distributions, access to pasture, presence of paddock or environmental enrichments, automatic milking systems).

**Results:**

When hay was included in the diet, higher feeding synchronization (93.7 ± 1.6 vs. 52.2 ± 4.7%; *p* < 0.01), percentage of cows with relaxed ear postures (35.8 ± 5.4 vs. 15.5 ± 2.1%; *p* < 0.01) and percentage of cows with no visible eye white (55.9 ± 17.0 vs. 36.6 ± 4.1%; n.s.) were recorded. A higher level of feeding synchronization was observed also when the feeding places/cow ratio was > 1 (72.1 ± 9.9 vs. 53.8 ± 5.8%), although differences were not significant (*p* = 0.14). Deep litter had a more positive effect than cubicles on comfort at resting, with a significantly higher percentage of ruminating cows (65.8 ± 10.2 vs. 34.2 ± 3.7%; *p* < 0.01), a higher percentage of cows with no visible eye white (55.6 ± 9.9 vs. 33.1 ± 3.7%; *p* < 0.05) and a higher percentage of cows in a more comfortable posture, with stretched legs (14.3 ± 5.1 vs. 5.6 ± 1.6%; *p* = 0.09). QBA highlighted the most positive emotional state in the only farm that allowed access to pasture.

**Conclusions:**

This study represents a first attempt to apply a protocol for on-farm welfare evaluation based exclusively on the use of positive welfare indicators and provides suggestions on possible benefits (e.g., deep litter, feeding places/cow ratio > 1, hay in the diet and access to pasture) to enhance dairy cattle welfare.

## 1 Introduction

The current European approach to animal welfare promotes the use of animal-based indicators to evaluate animal welfare (1) and considers husbandry systems and management procedures as factors that may have a positive or negative impact on animal welfare. Factors which are likely to cause negative effects on animal welfare are defined as “risks,” whereas factors which are likely to improve animal welfare are regarded as “benefits” (2). This is in line with the most recent views on animal welfare (3), which go beyond the traditional approach aiming to prevent suffering and include positive states and emotions that might be generated by positive experiences. This view lead to funding a specific European research network on positive welfare [“LIFT,” Lifting farm animal lives – laying the foundations for positive animal welfare, within the EU COST Action; (4)]. The absence of negative states *per se* is not sufficient to ensure high welfare standards, while it is essential that animals can have positive experiences during their lives. This is why the concept of positive welfare is rising in animal welfare science, and research is now moving from the need to guarantee the simple survival of animals and alleviation of suffering towards the concept of “a life worth living,” in which the balance between positive and negative affects is in favor of the former, and animals have the opportunity of undergoing rewarding experiences and fulfilling states (3, 5). Furthermore, promoting positive welfare is in line with consumers’ and citizens’ expectations, and can be successfully communicated to provide an added value to animal products (3, 6).

Despite the acknowledged importance of promoting positive welfare, few indicators are currently available to measure it in dairy cows (7). Among these, feeding and lying synchronization, eye aperture (eye white), ear posture, lying postures and Qualitative Behavior Assessment seem to be the most promising indicators for dairy cattle in terms of validity, as they seem to be able to highlight positive affective states; however, further research is required to assess the feasibility of some indicators and to identify the best strategy for data collection on farm (7). For example, feeding and lying synchronization are indicative of a strong cohesion (which is very important in social species, as cattle) and of reduced competitiveness within the herd (7, 8). Furthermore, a higher level of lying synchronization was found to be related to the provision of a higher amount of space and to the presence of comfortable and insulated lying surfaces (9). A comfortable housing situation also seems to allow cows to show their preferred lying postures, with their head tucked against the flank, or with outstretched legs (10, 11). In the same positive housing contexts, a higher percentage of cows ruminating while lying was also observed (10). Good levels of inter-observer reliability and consistency over time are reported for lying synchronization and for lying postures by Plesch et al. (12). As to eye white and ear posture, their validity has been confirmed in a study by Battini et al. (13), who found a decrease of the portion of visible white in the eye and a more relaxed ear posture (i.e., held backwards on the cows head, not passively drooping or upright, or hung down loosely with the ear pinna facing downwards) when cows were experiencing a positive situation. Unfortunately, no information about the reliability of these indicators is currently available in the published literature. Additionally, allogrooming and use of brushes have later been suggested as promising indicators (14). The absence of fear of humans is also considered an indicator of positive welfare, as it reflects the fact that animals have positive contacts with stockpersons. A valid indicator to assess the quality of human-animal relationship in cows is the Avoidance distance test which is included in Welfare Quality^®^ assessment protocol for dairy cattle (15) and shows a high to very high inter-observer reliability and a high intra-observer reliability (16). However, the Avoidance Distance measures the avoidance reactions of cattle to an approaching human, and it may therefore be interpreted as an indicator of negative feelings, i.e., fear. This is why in this study we did not consider the avoidance distance as variable, but we retained only the acceptance of human contact as indicator of positive subjective experience of the animals, based on previous studies (17) showing that increased voluntary stockperson contacts with the cows were associated with more positive emotional valence, evaluated using Qualitative Behavior Assessment (QBA). QBA is also included in the Welfare Quality^®^ assessment protocol for dairy cattle (15) and it has been used in several studies on dairy cows to describe how animals interact with the environment and to holistically highlight the emotional valence (from negative to positive) and the level of arousal (from low to high) in the herd, based on an integrative approach where the “whole-animal” is assessed using a free-choice profile or a pre-determined list of fixed terms, defined as “descriptors” (18, 19), and showed an intra-and inter-observer reliability ranging from strong to very strong on the first three components generated by Principal Component Analysis carried out on the descriptors (19).

This research presents preliminary results from a broader integrated project (“Development of an innovative supply chain management model to improve internal and external information flow, optimize processes and obtain sustainable and high-quality dairy products that meet consumers’ demand”) led by a cooperative of milk producers, aiming to define, develop and validate operational tools to undertake a virtuous path for improving the supply chain in relation to animal welfare, environmental sustainability, quality and traceability of the processes and products of the entire supply chain. This will be achieved by identifying and assessing few selected parameters that will be used as criteria to pay additional bonuses to virtuous farmers. As to animal welfare, the presence of benefits that are likely to enhance positive welfare will be rewarded. The aim of this research is to test a prototype protocol based exclusively on positive welfare measures in 20 dairy cattle herds, and to present some preliminary results which may help to identify potential benefits that are likely to promote positive welfare.

## 2 Methods

Positive animal welfare was assessed in 20 loose housing dairy cattle farms (6 on deep litter with straw, 14 in cubicles) located in the Po Valley (Northern Italy). Farms were selected among those delivering milk to a cooperative of milk producers, based on the availability of farmers to be assessed, and trying to reach a balance among all the considered housing and feeding factors described below. Unfortunately, given the limited possibility of choice, in spite of our efforts the sample was not balanced for all factors. Farms’ characteristics are reported in Table 1. In each farm we assessed one pen. When more than one pen was present in the farm, we chose the one presenting the potentially greatest risk for welfare in terms of higher density, lower feeding space/animal ratio, lower drinking place/animal ratio (20). If all pens were in similar conditions, one random pen was selected (excluding infirmary pen), and all the cows in the pen were evaluated; the number of cows evaluated in each pen is reported in Table 1. All the farms had sufficient water availability, according to the thresholds recommended by the Classyfarm National Welfare Assessment System (i.e., at least 1 drinker for every 10 animals or, if in a tub, 6–7 cm/head) (21).

**Table 1:** Characteristics of the 20 Italian dairy cattle farms.

Farm ID	N. lactating cows/ farm	N. cows evaluated/ pen	Breed	Housing system	Bedding material	Presence of mats	Cubicle size (length x width) (cm)	N. stalls/ cow^a^	m^2^ litter/ cow^b^	N. feeding places/ cow^c^	AMS	Diet	N. feed distrib.	Environ. enrich.^d^	Access to outdoor paddock	Access to pasture	Classyfarm welfare score^e^
A	45	45	HF	Cubicles	Straw	No	250×120	1.04	–	0.82	No	TMR	1	Yes	No	No	88
B	66	66	HF	Cubicles	Sawdust	Yes	215×125	1.14	–	1.38	Yes	TMR	1	Yes	No	No	73
C	24	24	HF	Deep litter	Straw	–	–	–	7.69	2.00	No	Hay + concentrate	3	No	Yes	No	78
D	135	73	HF + BS	Cubicles	Straw	No	232×120	0.96	–	0.75	No	TMR	1	No	No	No	79
E	80	80	HF	Deep litter	Straw	–	–	–	4.31	0.91	No	TMR	1	No	Yes	No	74
F	300	55	HF	Cubicles	Straw	Yes	250×125	1.31	–	1.00	No	TMR	2	Yes	Yes	No	88
G	32	32	HF	Cubicles	Straw	No	250×125	0.94	–	0.91	No	Hay	2	Yes	No	Yes	93
H	96	86	HF	Cubicles	Sawdust	Yes	214×110	1.12	–	0.95	Yes	TMR	1	Yes	No	No	75
I	180	80	CB	Cubicles	Straw	Yes	250×140	0.95	–	1.00	No	TMR	1	Yes	No	No	74
J	162	72	HF	Deep litter	Straw+ sawdust	–	–	–	6.94	0.36	No	TMR	2	No	Yes	No	81
K	190	90	HF	Cubicles	Compost	No	215×125	0.93	–	0.64	No	TMR	2	No	No	No	93
L	165	40	HF	Cubicles	Straw	No	250×125	1.00	–	1.00	No	TMR	1	Yes	No	No	83
M	63	63	HF	Cubicles	Straw	No	250×130	0.95	–	0.83	Yes	TMR	1	No	No	No	76
N	69	69	HF	Cubicles	Straw	No	215×100	1.04	–	0.84	No	TMR+ concentrate	3	Yes	Yes	No	88
O	15	15	HF	Deep litter	Straw	–	–	–	15.00	1.62	No	Hay+ concentrate	2	No	No	No	71
P	130	110	HF	Cubicles	None	Yes	225×125	1.05	–	0.82	No	TMR	1	Yes	No	No	73
Q	125	72	HF	Deep litter	Straw	–	–	–	4.88	1.04	No	TMR	2	No	No	No	NA
R	24	17	HF	Deep litter	Straw	–	–	–	7.06	3.35	No	TMR	1	No	No	No	72
S	72	72	HF	Cubicles	Sawdust	Yes	220×110	0.83	–	1.00	Yes	TMR+ concentrate	1	No	No	No	78
T	360	180	HF	Cubicles	Straw	No	210×130	0.78	–	0.52	No	TMR	2	Yes	No	No	84

HF, Holstein Fresian; BS, Brown Swiss; PRI, Italian Red Pied; CB, crossbreds.

a ≤ 1 stall/head: insufficient, > 1 stall/head: sufficient.

b ≤ 6.7 m^2^/head: insufficient, > 6.7 m^2^/head: sufficient.

c ≤ 1 feeding place/head: insufficient, > 1 feeding place/head: sufficient.

d Brushes; TMR, total mixed ration.

e NA, not available.

Feeding synchronization (22), resting synchronization (9), rumination during resting (8), and comfortable lying postures (11) were evaluated. The quality of the human-animal relationship was evaluated using the Avoidance distance at the feeding rack test (ADF test), with the percentage of cows that entered in contact with the assessor as response variable (15). Additionally, the animals’ emotional state during feeding and resting was estimated by assessing eye aperture and portion of visible eye white, and ear postures (13). QBA based on a predefined list of 20 fixed terms (15) was carried out to estimate the prevalent emotional state in the cows’ pen. Data collection started 2 min after the morning feed distribution and data was collected following a pre-defined order (Table 2). An accurate description of the methodology used to collect each welfare measure, including where, when, and how the indicators were collected, is provided in Table 2.

**Table 2:** Description of procedures for the collection of positive welfare measures.

Data collection	Where	When	How	Measures of positive welfare
Avoidance distance at the feeding rack test (ADF test)	Outside the pen	2 min after feed distribution	The observer stands in front of the animal at a distance of 200 cm, establishes a reciprocal visual contact with the animal, then starts to move slowly towards the animal at a speed of one step/s, 60 cm/step and the arm lifted with an inclination of 45°, the hand palm directed downwards, without looking into the animal’s eyes, and records the avoidance distance (AD), i.e., the distance when the animal shows the first avoidance reaction (moving backwards, turning or shaking its head). If the animal can be touched by the observer, AD is 0 (contact). The percentage of cows that enter in contact with the observer is calculated out of the total number of cows tested.	% of contact
Emotional state during feeding	Outside the pen	After ADF test	Photos of one random side of each cows’ heads, taken from a distance with a zoom lens (Canon EF 70–300 mm f/4–5.6 IS USM) while cows are feeding, and later classified for eye white (EW1: eye white clearly visible; EW2: eye white partially visible; EW3: normal eye aperture, no visible white; EW4: half-closed eye, no visible white) and ear posture (EP1: ear held upright with the ear pinna faced either forwards or rotated to the side; EP2: ear pinna directed forwards with the ear held horizontally; EP3: ear held backwards on the cow’s head, not passively drooping or upright; EP4: ear hung down loosely with the ear pinna facing downwards) following (13).	% of cows with no visible eye white (EW3 + EW4) during feeding % of cows with relaxed ear posture (EP3 + EP4) during feeding (Figure 1).
Observation of feeding synchronization	Outside the pen	Start within 15 min after feed distribution	Observation of the number of cows feeding at the same time (scan sampling at 5 min interval for 60 min)	Max % of cows feeding simultaneously
Qualitative Behavior Assessment (QBA)	Outside the pen	1 and a half hour after feed delivery	Observation of the way in which all the animals in the enclosure express their behavior. Observations last 10 min if all the animals can be observed from a single observation point, or 20 min divided by the number of observation points (i.e., if 2 observation points, point 1 for 10 min, point 2 for another 10 min). At the end of the observation, the observers move away from the enclosure that should no longer be observed and then they score the 20 descriptors from first to last without jumping from one to the other in a disorderly manner (15).	Scores of the QBA 20 descriptors (Active, Relaxed, Fearful, Agitated, Calm, Content, Indifferent, Frustrated, Friendly, Bored, Playful, Positively occupied, Lively, Inquisitive, Irritable, Uneasy, Sociable, Apathetic, Happy, Distressed)
Resting synchronization	Outside the pen	After QBA	Observation of the number of cows lying down at the same time (scan sampling at 30 min interval for 60 min)	Max % of cows lying down simultaneously
Comfort around resting	Inside the pen	Between the first and second scan for resting synchronization	Observation of each cow’s lying posture (head and legs posture), presence of rumination.	% of cows ruminating while lying down. % of cows with stretched legs (Figure 2). % of cows with head against the flank.
Emotional state during resting	Inside the pen	Between the first and second scan for resting synchronization	Photos of one random side of each cows’ heads, as for emotional state during feeding.	% of cows with no visible eye white (EW3 + EW4) during resting % of cows with relaxed ear posture (EP3 + EP4) during resting

**Figure 1:**
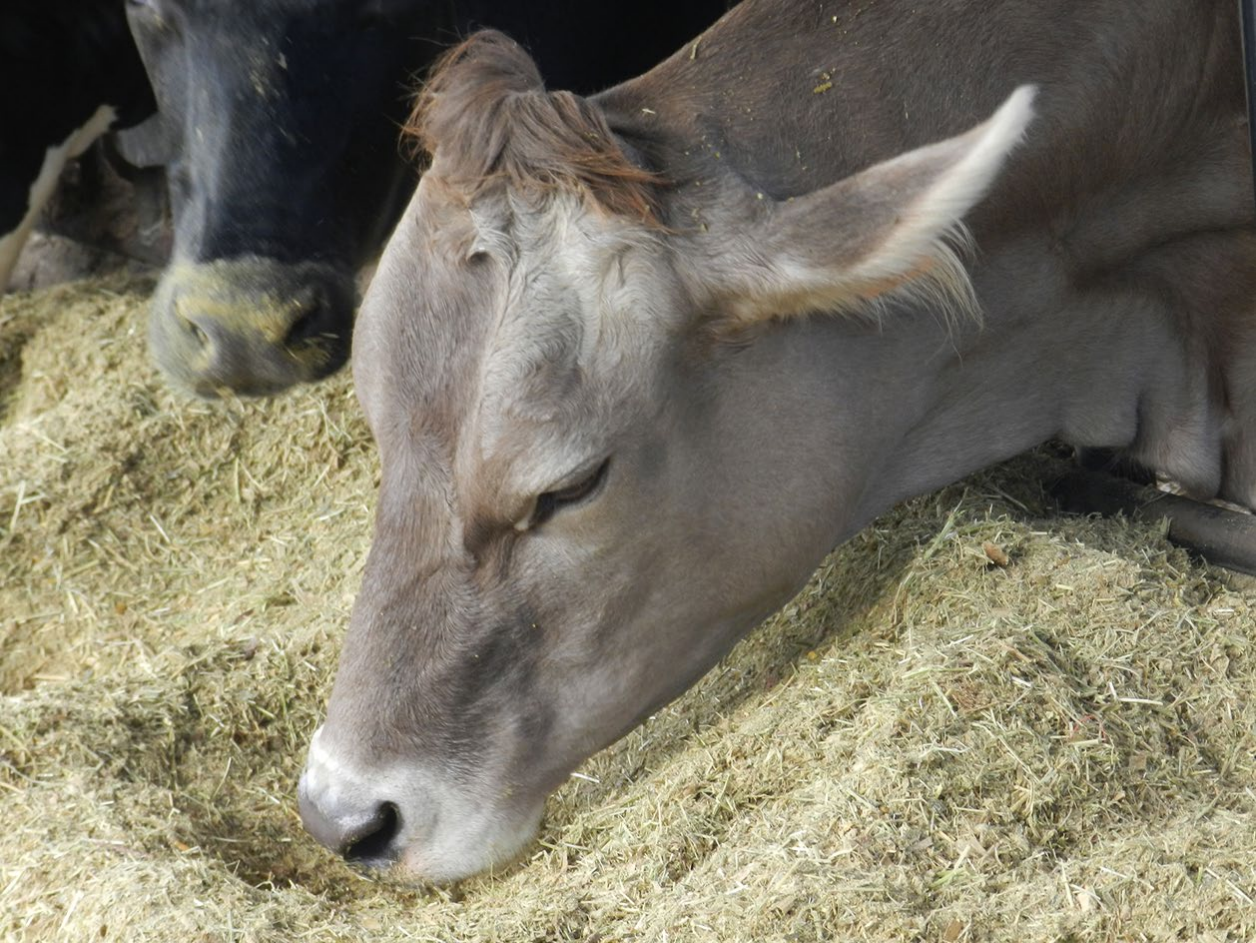
Cow in a relaxed mood while eating, with no visible eye white and a relaxed ear posture (ears held backwards on the cow’s head, not passively drooping or upright). For further examples of eye white and ear postures, please refer to Battini et al. (13).

**Figure 2:**
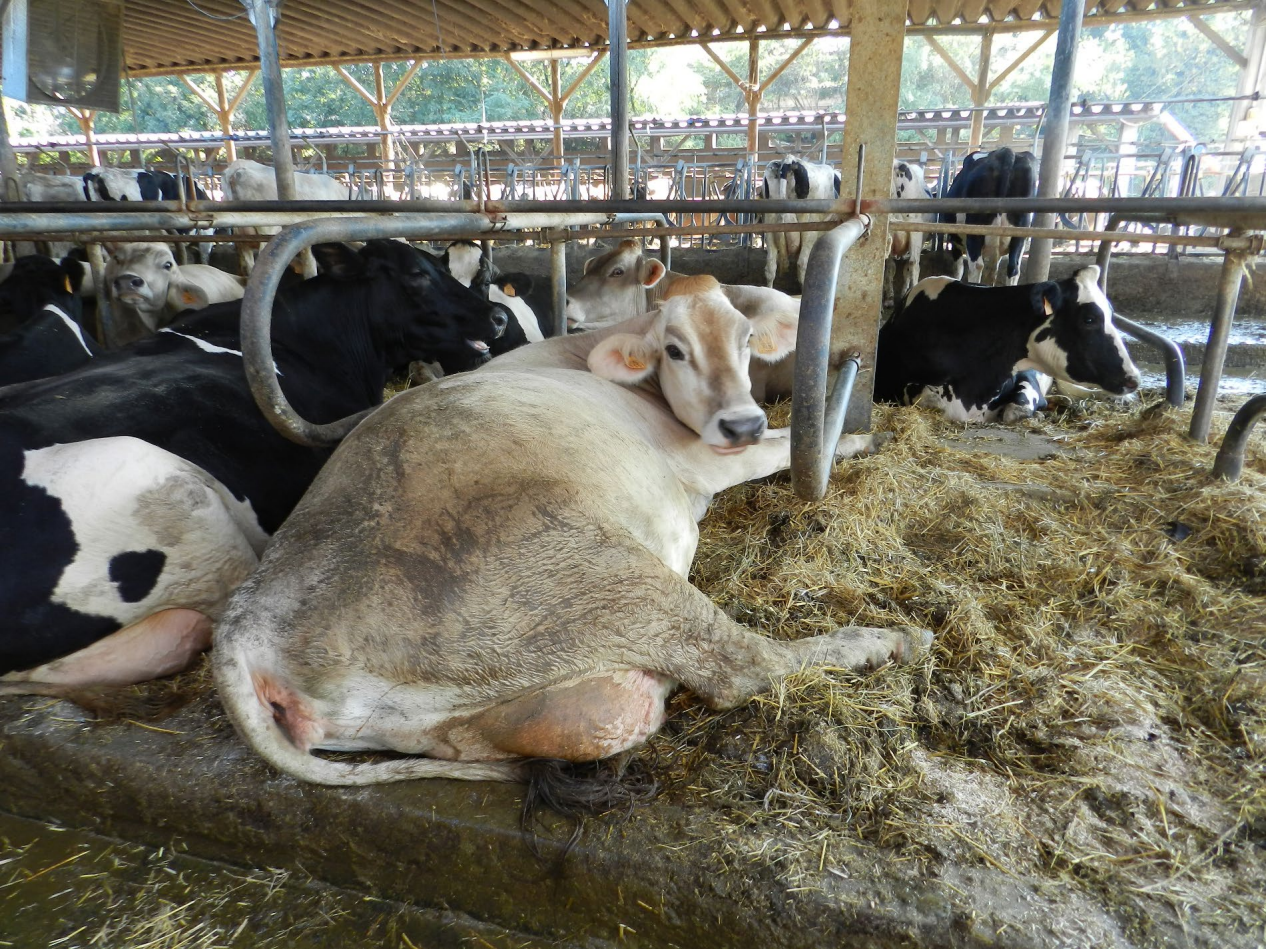
Cow in a comfortable resting posture, with stretched legs.

Data collection was performed by a trained assessor, who had previously collaborated to the development of the prototype protocol and who already had extensive experience on the recording of all the considered measures. For the indicators whose reliability had already been checked (QBA, synchronization, lying postures and ADF; see detailed information in the introduction section), we did not carry out further reliability analysis. For eye white and ear postures no information on reliability was available, therefore we checked the inter-observer reliability of two assessors scoring the same photographs from three sample farms, using the Gwet’s *γ(AC_1_)* agreement index, as suggested by Torsiello et al. (23) for the evaluation of inter-observer reliability between two assessors, when dealing with four-level variables. For ear posture, inter-observer reliability ranged from 0.72 to 0.86, whereas for eye white it ranged from 0.49 to 0.76.

As data was not normally distributed, the independent effects of housing [type of housing (deep litter vs. cubicles); sufficient (> 6.7 m^2^/cow on deep litter or > 1 cubicle/cow in cubicles) vs. insufficient (≤ 6.7 m^2^/cow on deep litter or ≤ 1 cubicle/cow in cubicles)] space availability (24); sufficient (> 1 feeding place/cow) vs. insufficient (≤ 1 feeding place/cow) number of feeding places/cow] and feeding [diet (presence/absence of hay or fresh grass in the diet) factors on relevant variables were compared by Mann–Whitney U test. Given that the criteria for assessing space availability (sufficient vs. insufficient) differed between the two types of housing, for space availability the same analysis was additionally carried out separately on two subsets of data, one for each the type of housing (deep litter and cubicles). Values are expressed as means ± S.E.

Given that only a limited number of farmers agreed to be included in the study, some additional factors (number of feed distributions, access to pasture, access to outdoor paddock, presence/absence of environmental enrichments, automatic milking systems – AMS) were not evenly distributed in our sample. The effect of these unbalanced factors was therefore explored only using QBA, following the Welfare Quality^®^ assessment protocol for dairy cattle (15), and the results were submitted to Principal Component Analysis (PCA; correlation matrix, no rotation).

Farm records (Average cows’ age, Average mortality rate, Average milk yield/year/cow, Average Somatic Cell Count) were obtained from farm registers.

All the farms, except one, are yearly evaluated by a veterinarian, using the Classyfarm National Welfare Assessment System (21). This system is designed to analyze and compare a large amount of information from different sources to categorize farms based on risks, in terms of animal welfare, biosecurity, consumption of antimicrobials, antimicrobial-susceptibility profiles. In relation to animal welfare, Classyfarm takes into account information about environment, structures, management, and risks, collecting resource-based information (e.g., space availability, litter amount and quality, etc.) as well as some animal-based information focused on traditional welfare indicators, such as BCS, cleanliness, lameness, presence of lesions, etc. The final welfare score for each farm is expressed on a scale ranging from 0 (insufficient) to 100 (excellent). Farms are considered in good welfare condition if the score is above 60, and very good if the score is above 80. The scores for our sample farms are reported in Table 1.

All the analysis were performed in SPSS V 29.0.1.0.

## 3 Results

The average values of all the recorded positive welfare measures, as well as of farm records, are reported in Table 3.

**Table 3:** Overall means ± S.E. of welfare measures during resting (comfort around resting and cows’ emotional state) and during feeding (feeding synchronization and cows’ emotional state), and of farm records.

Welfare and production measures	Unit	Means ± S.E. (Min–Max)
Cows in contact during ADF test	% of cows	7.30 ± 2.12 (0.00–30.00)
Cows with no visible eye white during feeding	% of cows	39.49 ± 4.35 (5.26–88.24)
Cows with relaxed ear posture during feeding	% of cows	18.55 ± 2.51 (4.76–45.45)
Max % of cows feeding simultaneously	% of cows	58.42 ± 5.21 (20.00–96.15)
Max % of cows resting simultaneously	% of cows	46.73 ± 3.22 (3.85–66.11)
Cows with no visible eye white during resting	% of cows	39.84 ± 4.48 (10.50–100.00)
Cows with relaxed ear posture during resting	% of cows	22.97 ± 2.86 (0.00–43.20)
Cows ruminating while lying down	% of cows	43.67 ± 5.08 (16.67–100.00)
Cows with stretched legs	% of cows	8.21 ± 2.03 (0.00–36.00)
Cows with head against the flank	% of cows	3.69 ± 0.99 (0.00–13.00)
Average cows’ age	years	5.48 ± 0.36 (4.00–10.00)
Average mortality rate	% of cows	3.95 ± 0.62 (0.00–11.11)
Average milk yield/year/cow (corrected milk)	kg of milk	9521.65 ± 381.16 (6100.00–12000.00)
Average Somatic Cell Count	cells/mL	233050.00 ± 16364.83 (100000.00–357000.00)

No significant differences were observed for farm records, resting synchronization (Max % of cows lying down simultaneously) and for the ADF test (% of contact) depending on the considered housing and feeding factors.

### 3.1 Housing

Significant differences were observed for some variables related to comfort around resting and to the cows’ emotional state during resting, depending on the type of housing (deep litter vs. cubicles) (Table 4). The housing system did not significantly affect the level of synchronization during resting, which was low both in deep litter and in cubicles (Table 4).

**Table 4:** Means ± S.E. of welfare measures during resting (comfort around resting and cows’ emotional state), depending on the type of housing.

	Unit	Deep litter (*n* = 6)	Cubicles (*n* = 14)	*p* value
Max % of cows lying simultaneously	% of cows	42.86 ± 8.39	48.39 ± 2.70	0.447
Cows with no visible eye	% of cows	55.65 ± 9.87	33.06 ± 3.75	0.033^*^
Cows with relaxed ear posture	% of cows	28.03 ± 6.72	20.80 ± 2.89	0.397
Cows ruminating while lying down	% of cows	65.84 ± 10.18	34.17 ± 3.69	0.005^**^
Cows with stretched legs	% of cows	14.27 ± 5.06	5.61 ± 1.63	0.091^§^
Cows with head against the flank	% of cows	2.42 ± 1.74	4.24 ± 1.21	0.353

§ *p* < 0.10; * *p* < 0.05; ** *p* < 0.01.

Space availability (sufficient vs. insufficient) did not significantly affect any of the considered variables, neither considering the whole data set (all farms), nor in the separate analysis carried out only in farms with deep litter, nor in the farms with cubicles.

**Figure 3:**
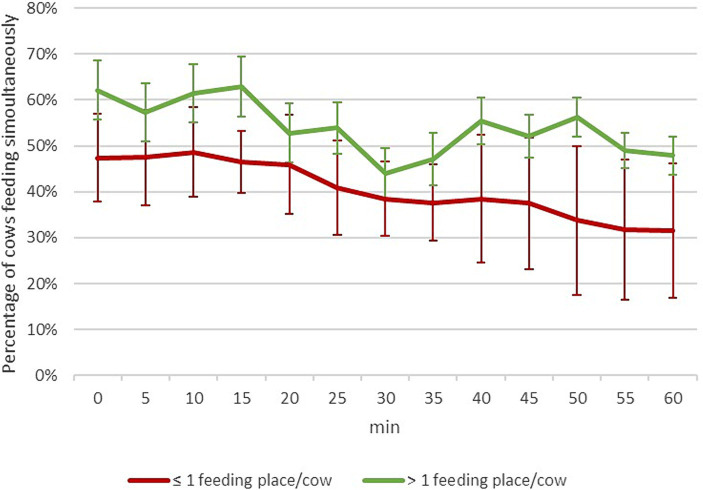
Effect of the number of feeding places/cow on the percentage of cows feeding simultaneously during the 60 min observation session started 15 min after feed distribution (scan sampling at 5 min interval).

The average level of feeding synchronization was always higher when the ratio feeding place/cow was sufficient (> 1 feeding place/cow) (Figure 3). The Max % of cows feeding simultaneously and the % of cows with relaxed ear posture were remarkably higher when the number of feeding places/cow was sufficient than when it was insufficient, although differences were not statistically significant (Max % of cows feeding simultaneously: 72.12 ± 9.88 vs. 53.86 ± 5.82% of cows, respectively; Cows with relaxed ear posture: 22.64 ± 6.36 vs. 17.19 ± 2.65% of cows, respectively).

### 3.2 Diet

The presence of long fiber (hay) in the diet positively affected the level of feeding synchronization and the emotional state of cows during feeding (Table 5).

**Table 5:** Means ± S.E. of welfare measures during feeding (feeding synchronization and cows’ emotional state), depending on the presence/absence of hay in the diet.

	Unit	Presence of hay (*n* = 3)	Absence of hay (*n* = 17)	*p* value
Max % of cows feeding simultaneously	% of cows	93.74 ± 1.63	52.19 ± 4.66	0.002**
Cows with no visible eye white	% of cows	55.91 ± 16.97	36.60 ± 4.07	0.358
Cows with relaxed ear posture	% of cows	35.82 ± 5.39	15.50 ± 2.07	0.012*

* *p* < 0.05; ** *p* < 0.01.

### 3.3 Other factors

**Figure 4:**
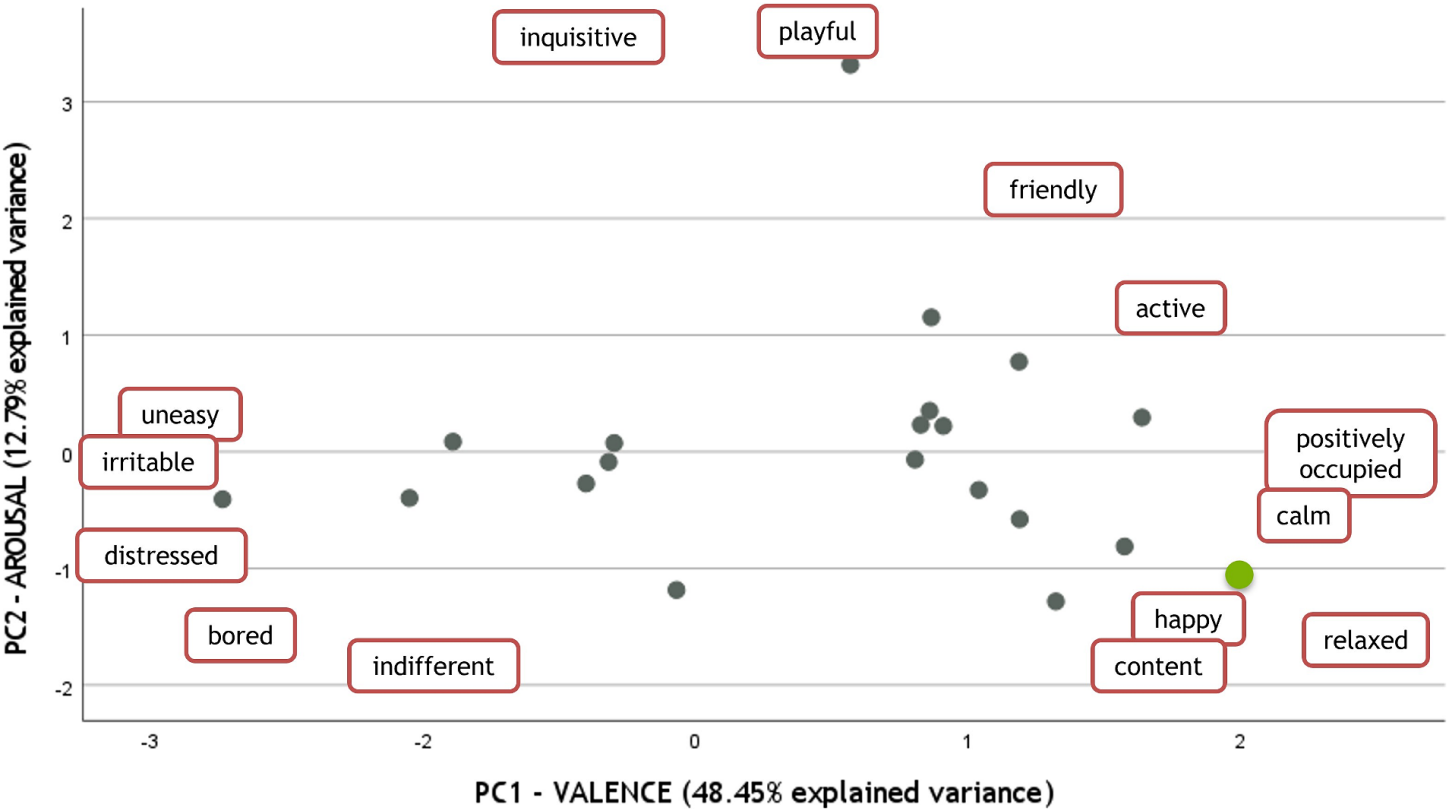
Loadings of the descriptors (in red frames) and scores of the farms (circles) resulting from PCA performed on QBA descriptors. The only farm where cows had access to pasture is highlighted by a green circle and is scattered in the bottom right corner of the plot, characterized by positive valence and low arousal.

The effect of other factors was explored using QBA, and results were explored by PCA. The first two Principal Components (PCs) explained 61.24% of the total variance (48.45 and 12.79% explained by PC1 and PC2, respectively). The distribution of the farms on the first two PCs was not affected by the number of feed distributions, presence of environmental enrichments, presence of outdoor paddock, nor presence of AMS. The farms were evenly distributed along the first two PCs, independently of the above-mentioned factors (data not shown). The only factor which seemed to affect farm distribution on the first two PCs was the possibility of access to pasture, which was allowed only in one farm (farm G, see Table 1). This farm had the highest scores on PC1, which correspond to the higher loadings of descriptors with a positive valence (friendly, active, positively occupied, calm, happy, content, and relaxed) (Figure 4).

## 4 Discussion

All the visited farms are typical intensive dairy farms of the Po Valley, where cubicles are the more common housing system; Holstein Friesian is the prevalent breed; production levels are generally medium-high; some farms have SCC values above the suggested threshold value suggested by Ruegg and Pantoja (25) for a healthy mammary quarter (<200,000 cells/mL), but always below the Italian legal requirements (<400,000 cells/mL); the average mortality rate is below the acceptable threshold defined by the Classyfarm National Welfare Assessment System (5%) (21), even though some alarming values above 10% are observed in a few farms.

Some problems were observed in relation to housing: only 11/20 farms (55%) allow cows with enough cubicles or a sufficient space on deep litter, and only 5/20 (25%) provide a sufficient proportion of feeding places/cow.

The average levels of activity synchronization are low, both for feeding (58%) and for lying (47%) (Table 3), compared to the levels suggested in the available literature, which range from 70% (26) to 90% (27), whereas the review by Napolitano et al. (28) reports a suitable threshold of 80%. The low level of lying synchronization might be justified by the time in which the observations were carried out, i.e., about 2 hours after the morning feed delivery (see Table 2). This moment of the day was chosen because we supposed that 2 hours after feeding the cows would start resting. However, recent work showed that the peak of lying synchronization in dairy cows mostly occurs between 2:00 and 6:00 a.m. (29), and this may explain why we seldom recorded a high synchronization level. Obviously, for on-farm welfare assessment it is not feasible to perform the observations during the night, and in Kok’s study (29) synchronization was actually measured by means of accelerometers, which in the near future can be regarded as a valuable tool for non-invasive monitoring of behavioral synchronization for the development of welfare monitoring schemes. Also measures of cows’ emotional state, as eye white and ear posture, provided scarce evidence of positive emotions (Table 3). During feeding this may be explained by the insufficient ratio between the number of cows and the number of feeding places, which according to Collings et al. (30) may enhance the level of agonistic behavior. In fact, more relaxed ear postures were observed when the number of feeding places/cow was sufficient, i.e., when all cows had access to at least one feeding place, which is assumed to be the basic standard (31). However, it is important to consider that feeding is a high-arousal activity, because cows are excited to access the feeding rack, and attentive ear postures (i.e., ears in upright position or directed forwards) are expected (13). Furthermore, more relaxed ear postures and a higher level of feeding synchronization were observed when hay was included in the diet, suggesting that cows appreciate this type of feed. According to the opinion of the European Food Safety Authority (EFSA), the quantity and quality of fiber in the diet should be regarded as an important factor that can affect cows’ anatomy, physiology, behavior, and health (32). In agreement with this opinion, our results suggest that the provision of long fiber, such as hay, can be considered as a benefit for dairy cows.

If high levels of excitement can be expected when cows are feeding, we would expect a higher proportion of animals with relaxed ear posture and no visible eye white when they are resting. Unfortunately, this was not the case in our sample, with some exception. In fact, a more relaxed emotional state was observed in cows on deep litter; in this housing system, cows’ comfort was also higher than in cubicles, with a higher percentage of animals ruminating while lying down and in a comfortable posture (i.e., with stretched legs), which are considered positive behaviors (7). This supports EFSA’s opinion (32) that deep litter is better than cubicles for cows’ comfort. In this sense, deep litter housing systems may be considered as an additional benefit.

The lack of differences between housing systems and between classes of space availability for resting synchronization was unexpected, given that differences in response to different housing and management systems (33, 34) and space availability (35) were reported in dairy cows and fattening bulls, respectively. The lack of significance in our study might be because, in general, cows’ comfort during resting was not optimal in our farms, and thus the synchronization was always below the thresholds suggested by various authors (26–28), but this could also be due to the fact that many other factors contribute to determine cows’ comfort during resting, such as the bedding system and hygiene (36), stall design and cleanliness (37) or cubicle’s size and bedding material (38). Unfortunately, due to our limited sample size, we could not test all possible combinations of space, cleanliness, bedding material, etc. These aspects need to be further investigated in the future.

Environmental enrichments in our farms consist of brushes that are present in half of the visited farms. Furthermore, 25% of the farms allowed cows to have access to outdoor areas, which in cows housed indoor may also be considered as a form of enrichment, that encourages physical exercise and provides access to fresh air, sunlight and other weather elements (31, 39). However, the presence of these enrichments had no significant effect on any of the considered variables, and QBA could not highlight any clear trend in farm distribution, depending on the presence/absence of environmental enrichments. This is in contrast with our expectations, given that environmental enrichment strategies usually have positive effects on animal welfare. This effect was previously investigated in cows using QBA (40), showing that animals provided with enrichments, such as novel objects or access to an outdoor area, were more content, relaxed and positively occupied, and less fearful and bored than animals without enrichments. In our case, brushes cannot be considered a novel object, as they were always present in the barns, and the access to an outdoor area was not a novelty either, because it was present all year round. Furthermore, the effectiveness of brushes as environmental enrichments depends on several factors, such as the number of brushes/cow, their location and distance from the food bunk, heat stress conditions or sanitary conditions of the cows [reviewed by Arnold and Dudzinski (27) and Napolitano et al. (28)]. Similarly, the characteristics of the outdoor paddock (size, shape, design, presence of facilities…) can also influence its effectiveness as environmental enrichment (31). Unfortunately, given our low sample size, we could not control for all these factors, which may have masked the positive effects of the enrichment. Further research is required to clarify the effect of environmental enrichments on positive welfare. However, the introduction of enrichments suggests an interest of farmers for promoting animal welfare.

According to EFSA AHAW Panel (31) and Mandel et al. (39), pasture is even more effective than outdoor paddocks to promote foraging and social behavior, and to provide visual and olfactory stimuli. Therefore, access to pasture is recommended to promote positive emotional states in dairy cattle (34), as well as in other ruminant species [e.g., goats; (41)]. In our sample, only one farm gave cows access to pasture, because this management practice is not widespread in intensive dairy cattle farms, which are typical of the Po Valley. Therefore, our results cannot be considered representative of the population of dairy cow farms. However, in line with the existing literature [e.g., (21, 28)], QBA highlighted a more positive affective state in this farm. This aligns with the present CAP strategic plans, which encourage and support the provision of longer periods of pasture rearing to improve animal welfare (42).

In our sample, AMS were present in 4 out of 20 farms (20%). This proportion is in line with the distribution in North-Western Italian dairy farms, where AMS are found 21.4% of the farms (43). AMS may significantly affect animal welfare and may have implications on the quality of the human-animal relationship and of cows’ emotional state. These effects depend on several factors, including farm management and the design of the AMS. Among the negative effects we can list the lower chance for the farmers to interact with their cows directly and physically (44). Moreover, the farmer could be induced to rely exclusively on technology to monitor cows, further reducing the frequency of interactions with them (45). On the other hand, the possibility to automatize the milking process may reduce labor and stress in humans, with possible positive effects also on their interaction with cows. The latter, being able to decide when to be milked, would also benefit from a reduction of the stress linked to a fixed milking schedule (46). Contrary to our expectations, in our study the presence of AMS had no effect on any of the considered variables, including those related to the quality of the human-animal relationship. Since cows’ welfare, and thus also their emotional state, can be considered as the sum of positive experiences over the sum of negative ones (47), the lack of effects on cows’ emotions may be explained by other transitory emotional experiences that have accumulated over time and which have not been considered in this paper.

No significant differences were observed for farm records (Average cows’ age, Average mortality, Average milk yield/year/cow, Average Somatic Cell Count) depending on any of the considered housing or feeding factors. As to the effect of the dietary frequency on milk production, our results are in line with other studies, in which no effect was observed depending on the frequency of feed distribution (48, 49). Regarding the impact of the type of feed administered to cows in terms of milk production, many factors should be considered, such as the dimension of feed particles, the amount of concentrate feed in the diet, the hay maturation stage, etc., which were outside the focus of the present study. In any case, the absence of the effect of the type of feed on the quantity of milk produced is in line with the results reported by Gislon et al. (50), who found that balanced diets, characterized by high-quality forages and a low intake of soybean meals, lead to milk productions comparable with those obtained by administrating a conventional corn silage-based diet.

In the present study no effect of the type of housing (deep litter vs. cubicles) on the hygienic quality of dairy cows’ milk in terms of Average Somatic Cell Count was observed. This was unexpected, since cows are usually dirtier in straw bedding and therefore the bulk milk somatic cell count is usually higher in deep litter than in cubicles (51), and can be explained by a proper management of straw litter.

This study represents a first attempt to apply a protocol for on-farm welfare evaluation based exclusively on the use of positive welfare indicators. According to our results, positive welfare is seldom achieved in our sample farms. However, according to the Classyfarm system, all the farms had a high overall welfare score, and eight out of 20 farms were scored as excellent (Table 1). This suggests that welfare evaluations carried out using traditional animal-based and resource-based measures, as the Classyfarm system, are able to highlight situations of good welfare in terms of lack of suffering, but they cannot guarantee that animals are experiencing positive feelings and are in a positive affective state. Therefore, the introduction of at least some positive welfare measures in the existing protocols could be useful to provide a better and more complete view of animal welfare. In the light of this consideration, it is worth underlying that some of the considered indicators (e.g., percentage of cows in contact during ADF test, percentage of cows with no visible eye white or with relaxed ear posture, percentage of cows ruminating while lying down) showed a wide range of variation across the different farms, and may therefore be considered as promising indicators to highlight different levels of positive welfare. However, the feasibility of these indicators for on-farm welfare assessment may be challenging: the application of the whole protocol was time consuming, as it took almost 1 day to collect all the measures, because we had to wait for specific moments for recording each measure (see Table 2). Furthermore, some measures required further processing. This was true especially for ear posture and eye white, which had to be manually scored from the photos after the field work. In the future, the development of automatic systems to detect these measures would greatly facilitate the evaluation of the emotional state of cows. Fortunately, Precision Livestock Farming technologies are rapidly progressing and preliminary results for the automatic analysis of facial expressions in both cattle and pigs are encouraging (52), and will certainly contribute to the development of feasible methods for on-farm monitoring of the affective state of animals.

We are aware of several limitations of the present study, first of all the limited sample of farms (*n* = 20) and the restricted possibility to choose the farms (due to the need to select farms from a pool of those that deliver milk to the cooperative and that were available to be assessed), which led to a limited variation of resources and management and to an unbalanced distribution of factors. For these reasons, it was impossible to check for the combined effect of the considered factors, and this is something that would certainly require further investigation in a more balanced sample. This implies that our conclusions are weak from a statistical point of view and can only be considered as a first step to foster future research for the development of assessment schemes in the emerging field of positive welfare. However, we believe that these preliminary results may provide suggestions on possible benefits to enhance cattle welfare and to promote positive affective states. Although based on a limited and unbalanced sample of farms, the outcomes deriving from the present research suggest that deep litter, a feeding places/cow ratio > 1, the presence of hay in the diet and access to pasture are likely to enhance dairy cattle welfare, and that they can be considered as possible benefits. Based on these findings, the cooperative of milk producers has now introduced these benefits in the evaluation system for calculating the milk price to pay to milk producers, who are thus encouraged to provide such benefits. This approach, aiming to reward virtuous farmers, is supposed to push other farmers to take care of positive welfare, and can therefore be considered as an example to follow for other cooperatives or for dairy industries, that are more and more interested in selling products that meet consumers’ requirements. In addition to this, the results are of interest for a wider audience, that could easily be reached by conveying this information using for example social media platforms, that could help to stimulate interactions and discussion on the topic (53).

## Data availability statement

The raw data supporting the conclusions of this article will be made available by the authors, without undue reservation.

## Ethics statement

Ethical approval was not required for this study, as no animal manipulation nor modification of the husbandry routine occurred and cows were maintained and observed in their usual housing system, under their common farming routine, throughout the whole study period. Written informed consent was obtained from the owners for the participation of their animals in this study.

## Author contributions

SM: Conceptualization, Formal analysis, Writing – original draft. SC: Data curation, Investigation, Writing – review & editing. FS: Data curation, Investigation, Writing – review & editing. MB: Conceptualization, Writing – review & editing.
